# Use of a miniature diamond-anvil cell in a joint X-ray and neutron high-pressure study on copper sulfate pentahydrate

**DOI:** 10.1107/S2052252521010708

**Published:** 2021-11-20

**Authors:** Giulia Novelli, Konstantin V. Kamenev, Helen E. Maynard-Casely, Simon Parsons, Garry J. McIntyre

**Affiliations:** a EaStCHEM School of Chemistry and Centre for Science at Extreme Conditions, University of Edinburgh, King’s Buildings, West Mains Road, Edinburgh EH9 3FJ, United Kingdom; b School of Engineering and Centre for Science at Extreme Conditions, University of Edinburgh, Erskine Williamson Building, King’s Buildings, Edinburgh EH9 3FD, United Kingdom; c Australian Centre for Neutron Scattering, Australian Nuclear Science and Technology Organisation, New Illawarra Road, Lucas Heights NSW 2234, Australia

**Keywords:** high-pressure study, neutron Laue diffraction, copper(II) sulfate pentahydrate, single-crystal neutron diffraction, single-crystal X-ray diffraction, materials science, inorganic chemistry, intermolecular interactions, crystallization under non-ambient conditions

## Abstract

Single-crystal X-ray and neutron diffraction data are usually collected using separate samples. This is a disadvantage when the sample is studied at high pressure because it is very difficult to achieve exactly the same pressure in two separate experiments, especially if the neutron data are collected using Laue methods where precise absolute values of the unit-cell dimensions cannot be measured to check how close the pressures are. In this study, diffraction data have been collected under the same conditions on the same sample of copper(II) sulfate pentahydrate, using a conventional laboratory diffractometer and source for the X-ray measurements and the Koala single-crystal Laue diffractometer at the ANSTO facility for the neutron measurements. The sample, of dimensions 0.40 × 0.22 × 0.20 mm^3^ and held at a pressure of 0.71 GPa, was contained in a miniature Merrill–Bassett diamond-anvil cell. The highly penetrating diffracted neutron beams passing through the metal body of the miniature cell as well as through the diamonds yielded data suitable for structure refinement, and compensated for the low completeness of the X-ray measurements, which was only 24% on account of the triclinic symmetry of the sample and the shading of reciprocal space by the cell. The two data-sets were combined in a single ‘XN’ structure refinement in which all atoms, including H atoms, were refined with anisotropic displacement parameters. The precision of the structural parameters was improved by a factor of up to 50% in the XN refinement compared with refinements using the X-ray or neutron data separately.

## Abstract 2

High-pressure single-crystal X-ray and neutron diffraction data for copper(II) sulfate pentahydrate have been collected under precisely the same conditions, using the same sample mounted in a miniature diamond-anvil cell. The data were combined in a joint ‘XN’ structure refinement.

## 1. Introduction

Coupling X-ray and neutron diffraction techniques provides the highest-quality structural information about a molecular solid in crystalline form (Finney, 1995 ▸; Schmidtmann *et al.*, 2014 ▸). In the past fifteen years, several studies have been performed towards obtaining joint neutron and X-ray analysis of samples under extreme conditions, at high pressure in particular (McMahon *et al.*, 2013 ▸; Guthrie, 2015 ▸).

Single-crystal X-ray and powder neutron diffraction experiments have been combined to study the effect of hydro­static compression on small organic molecules, such as the α and β forms of oxalic acid di-hydrate (Casati *et al.*, 2009 ▸; Macchi *et al.*, 2010 ▸), the ‘Y’ (yellow) polymorph of ROY and caprolactam (Harty *et al.*, 2015 ▸; Hutchison *et al.*, 2019 ▸). The two techniques have also been coupled in the study of a polymorph of glycolide recovered from high pressure (Hutchison *et al.*, 2015 ▸, 2017 ▸). Given the complementarity of X-ray and neutron diffraction data, ‘XN’ refinements have been performed with the aim of understanding high-pressure polymorphism in the amino acids l-serine (Wood *et al.*, 2008 ▸), l-serine monohydrate (Johnstone *et al.*, 2008 ▸), l-alanine and the aromatic amine aniline (Funnell *et al.*, 2010 ▸, 2013 ▸). In these joint refinements, the structural models used to understand the different diffraction experiments are linked together via common parameters describing the geometry of bond distances, angles, torsions and the thermal motions of the atoms.

The strong photon–electron interaction characteristic of X-rays, along with improvements in the design of diamond-anvil cells (DACs) and synchrotron technology, have led to the development of high-performance extreme-conditions beamlines, where molecular structures in the gigaPascal regime are solved and refined from single-crystal samples routinely (Moggach *et al.*, 2008 ▸; Nowell *et al.*, 2012 ▸; McMahon, 2015 ▸). Nevertheless, high-pressure data-sets, especially of low-symmetry crystals, suffer from low completeness because of shading by the DAC body, leading to low data-to-parameter ratios during refinement (Allan *et al.*, 2000 ▸; Dziubek *et al.*, 2007 ▸; Bergantin *et al.*, 2014 ▸). The problem of shading can be minimized by the use of focused high-energy synchrotron radiation and by merging data-sets collected individually from multiple differently oriented crystals in a DAC (Casati *et al.*, 2016 ▸). However, H atoms cannot usually be located in Fourier maps, meaning that they need to be placed geometrically or by employing a theoretical calculation (Giordano *et al.*, 2019 ▸; Novelli *et al.*, 2020 ▸). These limitations can lead to difficulties in thermodynamic modelling of intermolecular interactions and phase transitions, which depend critically on high-quality structural models and accurate H-atom positions.

Neutron data provide the nuclear positions in a crystal structure, with a much greater sensitivity to H atoms. Additionally, greater penetrating power permits the use of elaborate sample environments, such as the Paris–Edinburgh (PE) high-pressure press (Besson *et al.*, 1992 ▸). Previous work using powder diffraction methods has been referred to above, but these have some disadvantages. Peak overlap, which occurs because of powder averaging and which is exacerbated by strain broadening at high pressure, limits the complexity of the materials that can be investigated. The high background in neutron diffraction patterns caused by the large incoherent scattering cross section of the naturally abundant ^1^H isotope often requires the sample to be deuterated. However, deuteration can alter vibrational and other thermodynamic properties of a crystalline solid (Crawford *et al.*, 2009 ▸; Merz & Kupka, 2015 ▸), particularly near phase transitions, and can present a synthetic challenge even for quite simple compounds. Non-deuterated samples have been used in studies on gypsum (Henry *et al.*, 2009 ▸), glycolide and the polymorphic material ‘ROY’ (Hutchison *et al.*, 2017 ▸; Funnell *et al.*, 2019 ▸), but longer data-collection times are generally required to obtain statistics suitable for structure refinement. Ultimately, complementing high-pressure single-crystal X-ray data with high-pressure single-crystal neutron data would provide the captivating opportunity to study the same sample with two different diffraction techniques, if exactly the same conditions of temperature and pressure could be achieved.

Design and development of pressure devices for high-pressure single-crystal neutron diffraction have undergone rapid development in recent years. The VX PE press is used with both time-of-flight and monochromatic instruments (Klotz *et al.*, 2004 ▸; Bull *et al.*, 2009 ▸, 2011 ▸), and diamond/moissanite-anvil cells utilized with time-of-flight and steady-state Laue methods (McIntyre *et al.*, 2005 ▸; Bull *et al.*, 2009 ▸). Even so, none of these studies used a pressure device or a crystal of typical size suitable for laboratory X-ray diffraction measurements.

The first attempt at using a miniature DAC (mini-DAC) in high-pressure single-crystal neutron diffraction was reported by Binns *et al.* (2016 ▸). The experiment was made possible by Laue diffraction using the large solid-angle image-plate detector available on the VIVALDI diffractometer at the Institut Laue-Langevin and the Koala diffractometer at ANSTO (Cipriani *et al.*, 1996 ▸; McIntyre *et al.*, 2005 ▸, 2006 ▸; Edwards, 2011 ▸). The studies reported were performed on hexa­methyl­enetetramine and l-arginine di-hydrate at 300 and 150 K, the pressure was kept low (∼0.25 GPa) in order to validate the structural parameters against ambient-pressure data. The findings showed that diffraction data can be collected from an ‘X-ray-sized’ crystal in a mini-DAC with comparable completeness and resolution to those obtained with equivalent data collected at ambient pressure outside a pressure cell. Diffracted beams passing through the cell body were reliably measured and their intensities used in refinement, after correction for attenuation in the cell body.

A next-generation DAC with Versimax anvils, adapted for high-pressure neutron diffraction work at the Spallation Neutron Source (SNS) as well as the High Flux Isotope Reactor at the Oak Ridge National Laboratory (ORNL), have been reported by Haberl *et al.* (2018 ▸). The use of the cell for single-crystal studies on the high-symmetry magnetic materials MnP and Ba_0.4_Sr_1.6_Mg_2_Fe_12_O_22_, up to 8 and 4.7 GPa, respectively, was successfully demonstrated on SNS’s SNAP and CORELLI instruments as well as on the HFIR’s HB-3A and IMAGINE instruments. The DAC is also non-magnetic, opening the possibility for future studies under the combined extreme conditions of high pressure, low temperature and high-magnetic fields. A subsequent study by Massani *et al.* (2020 ▸) resolved deuterium positions in potassium di-deuterium phosphate (KD_2_PO_4_) and ice VI from single-crystal diffraction intensities collected on SNAP, at ambient-pressure and 1.1 GPa respectively.

A different DAC has been developed by Yamashita *et al.* (2020 ▸), with nano-polycrystalline diamond anvils and a bulk metallic glass (BMG) cylinder which is effectively transparent to neutrons and does not produce parasitic Bragg reflections. Ambient-pressure measurements in the DAC, using a single crystal of NaCl, on the D9 diffractometer at the Institut Laue-Langevin, showed that diffraction peaks can be measured without major angular restrictions and that the simple geometry of the cell allows accurate attenuation corrections. In the experiment on ice VII at 2.35 GPa, a single diffraction peak corresponding to the 110 reflection was observed before the BMG cylinder shattered.

Single-crystal neutron measurements at 1.0 GPa have been performed also by Grzechnik and co-authors in a piston–cylinder panoramic DAC with a monochromatic hot-neutron radiation source on the four-circle diffractometer HEiDi at the Heinz Maier-Leibnitz Zentrum (Grzechnik *et al.*, 2018 ▸). This study, carried out on the hexagonal magnetocaloric MnFe_4_Si_3_, demonstrated that the data collected are of very good quality and can be used for a full and reliable structural refinement. The same group investigated the material further in a subsequent combined X-ray and neutron single-crystal diffraction study performed in a transmission DAC at 0.9 GPa (Grzechnik *et al.*, 2020 ▸), the aim being the determination of the fractions for the mixed occupancy site incorporating Mn and Fe atoms. Their joint ‘XN’ structure refinement of the high-pressure synchrotron and neutron single-crystal data proved to be successful, as they unambiguously refined the occupancies of the two metals on the available sites. Fifteen unique reflections were measured in this study, due to the restrictions in angular range of the DAC, but completeness was 62%, aided by the high lattice symmetry of the sample.

Here, the potential of the ‘XN’ structure refinement has been extended to the study of a low-symmetry material. We have used single-crystal X-ray and neutron Laue data, collected on the same sample under identical conditions of pressure and temperature, to investigate the crystal structure of the triclinic salt hydrate copper(II) sulfate pentahydrate. This material holds a special place in the history of crystallography because it yielded the first recorded crystalline diffraction pattern by Friedrich, Knipping and von Laue (Friedrich *et al.*, 1913 ▸). Though it has remained a compound of interest ever since (Hume *et al.*, 1931 ▸; Beevers & Lipson, 1934 ▸; Taylor & Klug, 1936 ▸; Bacon & Titterton, 1975 ▸; Varghese & Maslen, 1985 ▸; Lipson, 1990 ▸), no work has been published to examine its response to pressure. While the atomic coordinates of O, S and Cu atoms can be obtained with relative ease from high-pressure X-ray diffraction, the high completeness and high sensitivity to H atoms characteristic of neutron Laue diffraction have been essential in order to locate them accurately.

## 2. Experimental

### 2.1. Crystallization

Pure, isotopically normal, copper(II) sulfate pentahydrate (CuSO_4_·5H_2_O) was obtained from Sigma–Aldrich. Blue, trapezoidal crystals were obtained from a warm (328 K) saturated aqueous solution, which was filtered and allowed to cool to room temperature. Crystals of suitable size for a mini-DAC loading were screened by acquiring single-crystal neutron Laue patterns on the Laue single-crystal diffractometer Koala on the OPAL reactor at ANSTO (Edwards, 2011 ▸), to select a crystal that gave sharp, intense Laue spots.

### 2.2. Data collection

#### 2.2.1. Mini-DAC loading

**Figure 1:**
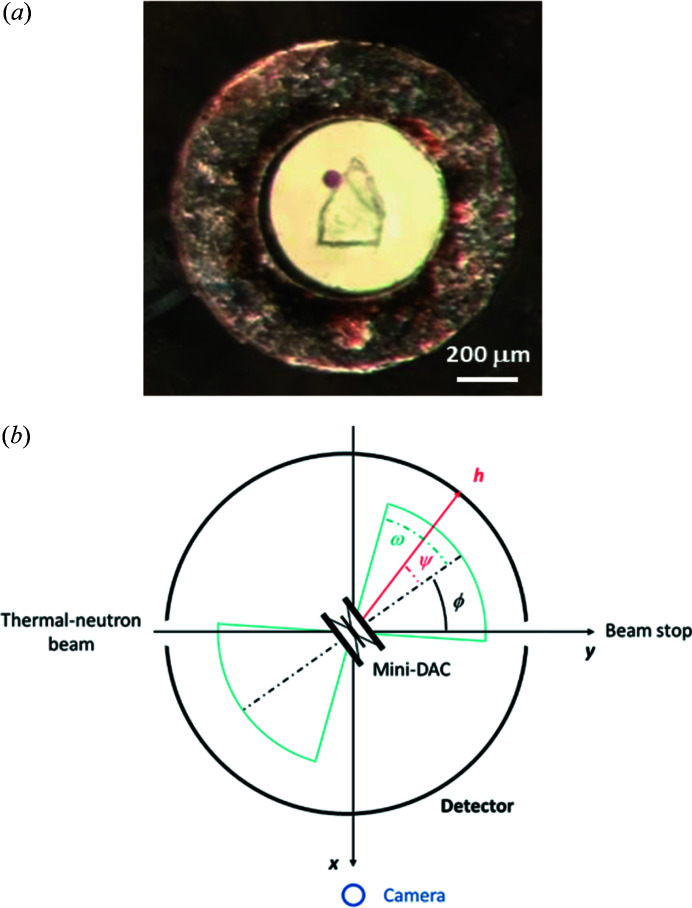
(*a*) Microscope photograph of the copper sulfate pentahydrate crystal with a chip of ruby in the mini-DAC gasket. (*b*) Geometry of the Koala instrument and mini-DAC view along the *z* axis, the latter being enlarged to show the geometry. The angle which the scattered ray, *h*, makes with the cell axis is denoted ψ.

A crystal of dimensions 0.40 × 0.22 × 0.20 mm was loaded in a Merrill–Bassett type mini-DAC with a half opening angle, ω, of 39° (Merrill & Bassett, 1974 ▸), Boehler–Almax-cut diamonds 3 mm in height with [001] culets of 1 mm diameter set into beryllium–copper alloy (BERYLCO-25) backing plates by 1.5 mm [Fig. 1 ▸(*a*)]. A description of the mini-DAC construction has been reported previously (Moggach *et al.*, 2008 ▸; Binns *et al.*, 2016 ▸). The gasket was constructed of 316 stainless steel, fully hardened with a yield strength of 0.9 GPa, but not pre-indented. The thickness of the gasket was 250 µm and a 700 µm hole was drilled to form the sample cavity. The hydro­static medium was a mixture of isotopically normal 1:1 pentane:iso­pentane (Klotz *et al.*, 2009 ▸) and a small ruby chip was used as a pressure marker, with the ruby fluorescence method applied to measure the pressure (Mao *et al.*, 1978 ▸).

In the configuration described, finite element analysis indicates that the cell has a maximum pressure of 5.4 GPa. The limiting factors are the materials used for the gasket and backing plate and the diamond anvil itself; the limit could be increased to 10 GPa with 800 µm culets and an indented BERYLCO-25 (rather than steel) gasket.

#### 2.2.2. Single-crystal neutron diffraction

The Koala Laue single-crystal diffractometer, a copy of the VIVALDI instrument at the Institut Laue-Langevin (McIntyre *et al.*, 2006 ▸), is situated at the end of a thermal-neutron guide on the OPAL reactor at ANSTO (Edwards, 2011 ▸). A cylindrical detector, consisting of a neutron-sensitive image plate, is used to record the diffracted Laue spots within a solid angle of 288° by 104°. In the orthogonal instrument coordinates system, the mini-DAC is at the origin, the vertical *z* axis points upwards, the *y* axis lies on the trajectory of the thermal-neutron beam, while the *x* axis completes a right-handed set. A simplified scheme of the Koala and mini-DAC geometries is shown in Fig. 1 ▸(*b*); a more detailed description is available in the work by Binns *et al.* (2016 ▸).

The mini-DAC was mounted within the helium closed-cycle refrigerator (CCR) available for the Koala diffractometer. A mounting screw glued to the cell body and a washer were used to place the cell at the correct height, the latter determined earlier using a spherical ruby crystal of 2 mm diameter. Even though this experiment on the sample was performed successfully at ambient temperature, the instrument was set up with a CCR to enable cooling, should this have been necessary (Binns *et al.*, 2016 ▸).

A ^6^LiF incident-beam aperture of 1 mm diameter, 229 mm from the sample centre, was used in addition to the standard borated-aluminium aperture of 3 mm, 486 mm from the sample centre, to avoid the incident beam striking the cell. The alignment on the centre of the gasket hole was performed optically using a telescopic camera with a telescope/monitor magnification of ×7, the contrast being too poor to allow the sample to be seen. Initially, the cell axis was rotated along the vertical *z* axis to reach a through-the-diamonds view, which allowed the adjustment of the sample height and offset along the *x* axis. Then, a view through the gasket was needed to adjust the offset along the *y* axis and make sure that the initial angle of rotation (ϕ) made by the cell axis and the *y* axis was approximately equal to 0°.

Three orientations of the mini-DAC, related by an approximate 120° rotation about the cell axis, itself perpendicular to the vertical instrument *z* axis, were needed to optimize completeness (see Section 2.2.3 for more details). For each orientation, Laue patterns were collected and related by motorized step-wise ϕ angular rotations, with |ϕ| ≤ ω to maximize flux at the sample. Additional diffraction patterns were taken with the incident thermal-neutron beam passing through the gasket, at ϕ ∼ −90°, because of the low-symmetry of the sample (space group *P* 1). The exposure time was between 10 and 11 h per Laue pattern. Data collection details are provided in Table 1 ▸.

The pressure-induced deformation of the near-perfect anvils reduces their primary extinction while transforming the strong diamond reflections into significant secondary sources, which give rise to tertiary powder rings from the cell body (Loveday *et al.*, 1990 ▸). The presence of such pseudo-Kossel lines in the Laue patterns indicated that the mini-DAC chamber remained sealed throughout the data collection and at a pressure of at least ∼0.25 GPa. The small variation (<1.5σ) in the mean of the ratios of refined lattice parameters for all the orientations demonstrates that the pressure remained constant throughout the experiment. Following data collection, the pressure was measured as 0.71 GPa.

#### 2.2.3. Reciprocal-space coverage in Laue geometry using a mini-DAC

**Figure 2:**
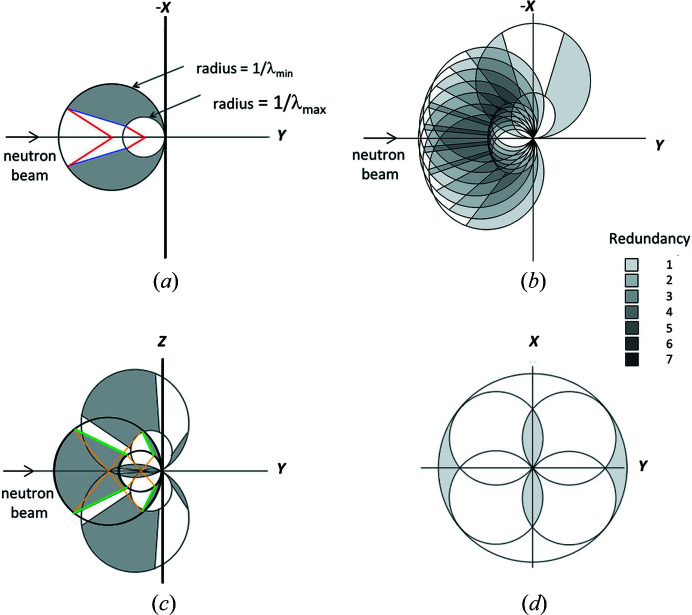
(*a*) Observable excited area in the equatorial plane for φ = 0°, with *x*, *y* and *z* (pointing out of the page) being the instrumental orthogonal coordinates system. The accessible areas of reciprocal space are dark grey in all insets. (*b*) Ewald construction in Laue geometry in the equatorial plane for typical high-pressure data collections using the mini-DAC. (*c*) Ewald construction in Laue geometry in the vertical instrumental *yz* plane (with *x* pointing out of the page) given a reorientation by ±120°, as possible with the mini-DAC. In this instance, the blind regions can be attributed to the erasing lamps, the incident- and transmitted-beam holes are ignored. Note that covered areas in (*b*) and (*c*) could be reflected through the origin, according to Friedel’s law. However the figures would be much more cluttered and therefore their reflections are omitted for the sake of clarity. (*d*) Ewald construction in monochromatic geometry in the equatorial plane for typical high-pressure data collections using a DAC with an opening half angle = 39° (cell axis is along *y*), assuming that the cell body is shielded. Entry of the incident beam from both sides of the cell is shown.

In a typical ambient-pressure neutron Laue diffraction experiment performed on the Koala instrument, three to twelve diffraction patterns are collected at different ϕ angles of rotation about the instrument’s vertical *z* axis, with Δϕ = 30 to 15°. For ‘new’ or large unit-cell samples Δϕ = 15–20° is usually chosen, if the material has low symmetry even smaller steps are desirable to improve the empirical wavelength normalization. The Ewald construction in Laue geometry in the equatorial plane is shown in Fig. 2 ▸(*a*) for ϕ = 0°.

The red lines in Fig. 2 ▸(*a*) indicate the 72° blind arcs on the Ewald ‘circle’ due to the erasing lamps for the two limiting λ_min_ and λ_max_ wavelengths. The blue lines indicate the limits of the blind area resulting from the combination of the blind arcs at all wavelengths. The reciprocal-space coverage, which is represented in dark grey, is reduced in the out-of-equatorial planes by sinχ, where χ is the out-of-plane angle around the incident thermal-neutron beam. All reciprocal lattice points that lie between the circles can give rise to observable Bragg reflections.

The cell of the mini-DAC body does not fully obscure the detector in high-pressure single-crystal neutron Laue diffraction, given that diffracted beams are able to travel through it providing additional useable data (Binns *et al.*, 2016 ▸). Fig. 2 ▸(*b*) shows the Ewald construction in a typical high-pressure data collection using the mini-DAC for ϕ = −36, −24, −12, 0, 12, 24, 36, 90°. This data collection strategy, which is similar to that applied for each orientation in this study (see Table 1 ▸), provides almost-complete reciprocal-space coverage, with some areas sampled up to five times. The addition of the Laue pattern at ϕ = 90°, collected with the thermal-neutron beam passing through the gasket, increases only slightly the completeness in the equatorial plane.

One orientation of the mini-DAC is sufficient for high-symmetry materials, while for triclinic crystal structures three orientations of approximately 120° around the normal to the cell axis, itself perpendicular to the vertical instrument *z* axis, are necessary to optimize completeness in the instrumental *yz* plane. Fig. 2 ▸(*c*) shows the cumulative reciprocal-space coverage in the vertical plane with a reorientation of the sample by +60 and −60°, equivalent to reorientations by +120 and −120°, as possible with a mini-DAC. The yellow lines indicate the ±52° (out of the equatorial plane) observable arcs on the Ewald ‘circle’ defined by the image-plate-free top and bottom ‘surfaces’ of the detector drum for the limiting wavelengths. The green lines indicate the boundaries of the blind area resulting from the combination of the blind arcs at all wavelengths. In three dimensions, the blind volumes caused by the top and bottom of the detector drum are elliptical cones with major and minor semi angles of 33.5 and 19°, respectively. Note that Friedel’s law, which even holds for most non-centrosymmetric crystal structures in neutron diffraction, allows the inversion through the orthogonal-system origin, adding more coverage at low *Q* (close to the centre).

This sampling of reciprocal space far exceeds that reported by Grzechnik *et al.* (2020 ▸). Fig. 2 ▸(*d*) shows a reciprocal-space-coverage schematic for a shielded DAC in a monochromatic experiment, which considers the equatorial plane of a DAC with an opening half angle of 39°. It is important to note that the completeness of the real experiment would not be the same as the percentage of the shaded area due to rotation of the figure around the *y* axis (to generate a volume), the real limit in 2θ on the particular diffractometer, and the limit of observability. With all the caveats, and in the specific case of the low-symmetry copper sulfate pentahydrate sample, a completeness of 24% is believable.

#### 2.2.4. Single-crystal X-ray diffraction

Single-crystal diffraction data were collected for the sample at 0.71 GPa and 295 K on an Agilent SuperNova diffractometer using Mo *K*α radiation (λ = 0.71073 Å) at the University of Sydney. Both the high-pressure neutron and X-ray diffraction experiments were performed under identical conditions of temperature and pressure, using the same cell and loading. A delay of 19 days between the two experiments was needed to wait for the activation of the mini-DAC to decay to a level that allowed removal from the neutron guide hall.

### 2.3. Data processing

#### 2.3.1. Single-crystal neutron diffraction

**Figure 3:**
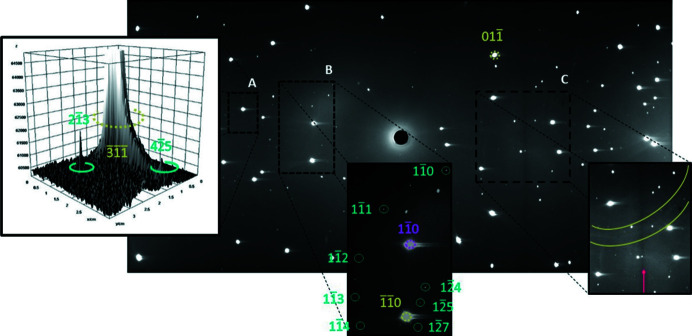
Laue diffraction pattern of copper sulfate pentahydrate recorded at 0.71 GPa, φ = −30°, using the mini-DAC. The pattern is dominated by scattering from the two diamond anvils. Inset A shows a surface plot of the reflection of one anvil (green) and its elongated tail due to residual fluorescence in the detector, the intensity being extremely high compared with that of the 213 and 425 sample reflections (light-blue). Inset B shows eight reflections coming from the sample, reflection 1 10 of one anvil (green) and reflection 110 of the other anvil (purple). The contrast was adjusted in the insets for the sake of clarity. Inset C represents the pseudo-Kossel line centred on the 011 reflection (green) and a vertical shadow cast by the gasket (magenta).

Laue diffraction data collected on the Koala instrument were visualized using *ImageJ* (Schneider *et al.*, 2012 ▸) and processed using the *LaueG* suite of programs (Piltz, 2018*a* ▸). A combination of intense reflections from the two anvils, pseudo-Kossel lines, vertical shadows cast by the gasket and residual traces of diamond spots from the preceding Laue patterns, gave rise to areas of locally increased background in the diffraction patterns that made the sample spots difficult to locate (Fig. 3 ▸). Previous tests have shown that the ruby crystal used as the pressure marker is too small to produce any significant Laue spots, even after 21 h of exposure time. A similar test was also performed on the gasket material outside the cell, without pre-indentation, and showed no Laue spots (Binns, 2016 ▸).

The two sets of diamond peaks were selected manually, indexed and related to one another by rotation of the crystal lattice (Fig. S1 of the supporting information). The procedure was applied to each pattern together with a refinement of the orientation offset to account for the diamonds orbiting around the centred sample. The indexed diamond reflections were used as a control for the rejection of outliers in the subsequent data analysis. The sample in each Laue pattern was indexed manually via an iterative procedure, starting from strong reflections aligned along arcs and then using the calculated spots as a guide for adding further sample Laue spots. The first pattern from each mini-DAC orientation was indexed from scratch. *LaueG* adjusted automatically the indexing for the change in ϕ of subsequent patterns with the same mini-DAC orientation. Comparison of the refined horizontal (projections of the real offsets in *x* and *y*) and vertical (along *z*) offsets over the full data-set confirmed the sample had been centred to within 0.10 (16) and −0.01 (30) mm horizontally and vertically, respectively. These values were found to be in good agreement with those obtained from the reference ruby crystal (0.17 and −0.07 mm, respectively). This rapid centring procedure differs from that used in previous experiments on higher-symmetry crystal structures (Binns, 2016 ▸), where the centre was chosen among a set of nine or more 1 h exposures with different *x*, *y* and *z* offsets by monitoring the intensity of the ‘presumed’ strong sample reflections. Though it worked well in those situations, parasitic spots of different origin may be easily mistaken for sample reflections, leading to a loss of instrument time.

**Figure 4:**
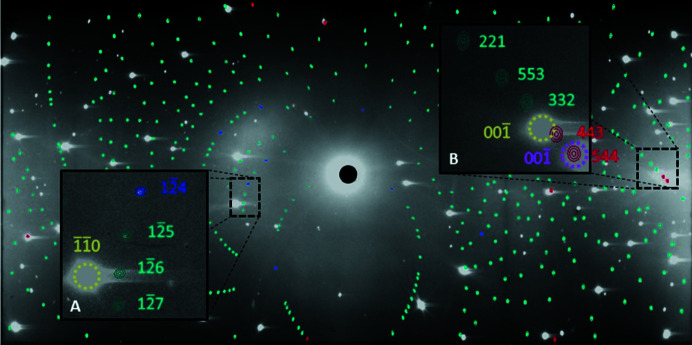
Integrated Laue diffraction pattern of copper sulfate pentahydrate recorded at 0.71 GPa, φ = −30°, using the mini-DAC. The blue pattern shows the sample ellipses, the darker ones being the model peaks (inset A). Inset B shows rejected sample Laue spots (red) that are very close to or overlapping the diamond reflections. The same colour code and contrast adjustment as those of Fig. 3 ▸ were applied.

Sample reflection intensities were integrated using a two-dimensional adaptation of the three-dimensional σ(*I*)/*I* algorithm from the literature (Wilkinson *et al.*, 1988 ▸; Prince *et al.*, 1997 ▸) which is implemented in *LaueG* with the program *argonne_boxes*. All strong Laue spots, both single and multiple, were used to make the library of model peak shapes that were then applied to the weaker reflections. The same set of parameters was applied to all Laue diffraction patterns (Table S1 of the supporting information), which were integrated in batch mode. The crystallographic resolution limit of 0.8 Å was determined iteratively by finding the minimum *d*-spacing at which ∼10% of the integrated reflections had *I*/σ(*I*) ≥ 5. Cross-checking between the predicted diamond reflections from the indexing and the sample ellipses produced by the integration showed that the program rejected potential model reflections on anvil spots. Additionally, ‘admissible’ sample spots very close to anvil spots were also rejected automatically on the basis of anomalies in the calculated background (Fig. 4 ▸).

**Figure 5:**
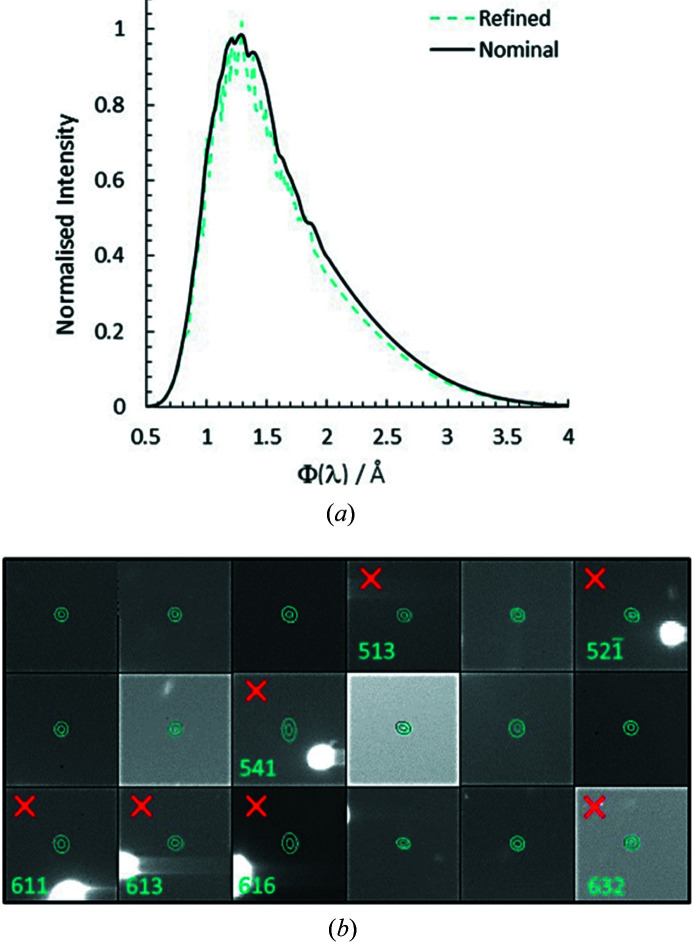
(*a*) Refined instrument wavelength spectrum for Laue data collected at 0.71 GPa for copper sulfate pentahydrate. The nominal spectrum is included for comparison. (*b*) Selected set of outliers produced during the normalization process, the red crosses indicating the sample Laue spots rejected manually given their proximity to diamond reflections or diamond tails.

The diffracted reflections passing through the mini-DAC body or gasket required an absorption correction, unlike those passing through the diamonds, their attenuation being determined by their wavelength and the path length through the cell or gasket (Binns *et al.*, 2016 ▸). The data were empirically normalized to a common incident wavelength within *LaueG* using the normalization routine *Laue4* (Piltz, 2018*b* ▸), where repeated observations (at different wavelengths) and equivalent reflections with wavelengths between 0.78 and 2.00 Å are compared [Fig. 5 ▸(*a*)]. High-pressure data-sets usually need an extended wavelength spectrum (conventional analysis is performed typically within the 0.80–1.75 Å range) to compensate for the lower number of well measured reflections. This can lead to higher values of the merging *R*-factor (*R*_int_) but also adds useable reflections to the final refinement. Crystal absorption and extinction corrections were deemed unnecessary because of the very small size of the sample. The normalization process was performed iteratively in order to optimize the merging statistics; the outliers were visually inspected and removed if situated too close to diamond reflections [Fig. 5 ▸(*b*)]. By allowing the incident beam spectrum to refine, the majority of the diffracted-beam events and diamond dips were removed as outliers of lower intensity during the normalization procedure. In all, just 1.3% of the observed single reflections were removed.

#### 2.3.2. Single-crystal X-ray diffraction

High-pressure data were indexed and integrated up to 0.70 Å using *CrysAlis PRO* (Agilent, 2014 ▸). Correction for the mini-DAC shading, absorption and other systematic errors was also applied using the multi-scan procedures available within the program.

## 3. Results and discussion

Individual refinements of the X-ray and neutron data were performed initially to check their internal consistency.

### 3.1. Refinement against X-ray data

Single-crystal X-ray diffraction data were refined by full-matrix least-squares on |*F*|^2^ in *Crystals* (Betteridge *et al.*, 2003 ▸), starting from the atomic coordinates of the ambient-pressure structure in the setting published by Varghese & Maslen (1985 ▸) [Inorganic Crystal Structure Database code 60059 (Hellenbrandt, 2004 ▸)]. Three different weighting schemes were tested, and are given in Table 2 ▸. Weighting scheme 1 was used in *SHELXL* (Sheldrick, 2015 ▸), where the parameters *a* and *b* were optimized in an analysis of variance. Weighting scheme 2 was the same except that *a* was fixed to 0.03 and *b* to 0, the values suggested by previous works (McCandlish *et al.*, 1975 ▸; Lundgren & Liminga, 1979 ▸). Weighting scheme 3 consisted of statistical weights [*w*_i_ = 1/σ^2^(*F*_o_^2^)] with the robust-resistant modifier given by Prince & Nicholson (1983 ▸).

Cu and S atoms were refined with anisotropic displacement parameters, while O atoms were refined isotropically. H atoms, which could not be located with confidence from difference maps, were placed at 0.84 Å from an O atom along a hydrogen-bond vector, giving an ∠H—O—H angle range between 96.5 and 131.4°. H-atom positions were not refined but updated iteratively until convergence. No restraints were applied. The *R*_1_-factor {*R*_1_[*F* > 4σ(*F*)]} was between 0.0601 and 0.0620, depending on the weighting scheme, with a data/parameter ratio of 9.2 and a completeness of 24%. The completeness is low even by the standards of high-pressure structure determinations because of the low symmetry of the crystal structure [See Section 2.2.3 and Fig. 2 ▸(*d*)].

**Figure 6:**
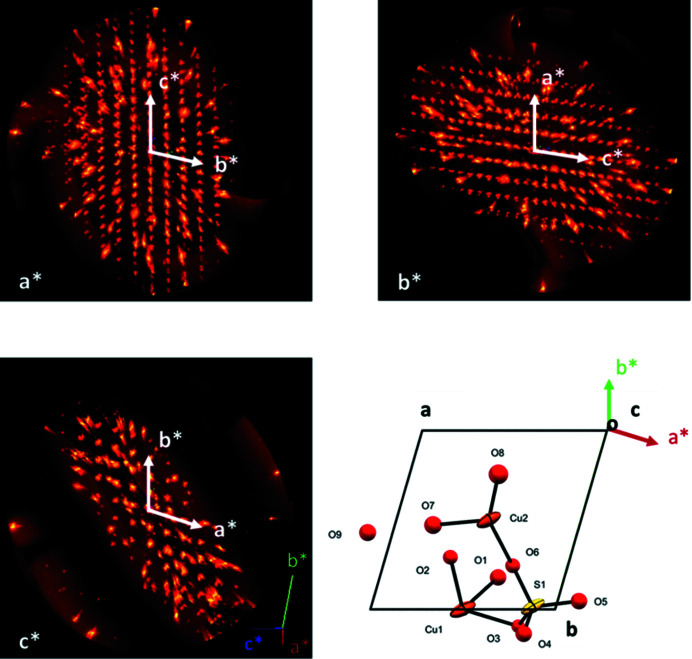
Reconstructed X-ray precession plots viewed along a*, b* and c*. The bright spots exhibiting radial elongation arise from the diamond anvils. The view along the c* axis clearly shows dark areas of reciprocal space due to the shielding of the mini-DAC. A view of the asymmetric unit of copper sulfate pentahydrate along the *c* axis shows the Cu—O and S—O bonds, with the H atoms removed from the structure for clarity, and helps to identify those bonds which have their projections predominantly in the a*b* plane.

The large variation of the estimated standard deviations of the Cu—O and O—S bond lengths (Table 3 ▸) is a consequence of the reduced reciprocal space covered in the X-ray data collection with the mini-DAC. Fig. 6 ▸ shows the experimental coverage viewed along the a*, b* and c* axes and a view of the asymmetric unit of copper sulfate pentahydrate along the *c* axis. Cu—O and S—O bond lengths with the higher estimated standard deviations are those lying predominantly in the a*b* plane, which corresponds to the least-explored area of reciprocal space.

### 3.2. Refinement against neutron data

A similar procedure was applied to the single-crystal neutron Laue diffraction data. The atomic coordinates used to initiate refinement were taken from the X-ray model. The unit-cell parameters were set equal to those determined in the X-ray study. All atoms, including H atoms, were refined anisotropically and without restraints, giving a final *R*_1_-factor between 0.1654 and 0.1760, a data/parameter ratio of 5.0, and a completeness of 68% (Table 2 ▸). Weighting scheme 3 was found to perform better than the other schemes, having the lowest estimated standard deviations on the geometric parameters, but this was achieved by setting the weights of 8.2% of the reflections to zero. This scheme down-weights data with high values of Δ|*F*| = |*F*_o_| − |*F*_c_|, effectively assuming that the model is broadly correct and that the values of *F*_c_ are close to their ‘true’ values.

Analysis of the un-merged contributors to the down-weighted reflections showed them to be localized in areas of highly varying background (see Figs. S2 and S3, Table S2). Therefore, the data-set was re-merged in *SORTAV* using the robust-resistant modifier suggested by Blessing (1997 ▸) and the refinement was repeated, yielding a completeness of 70% and a data/parameter ratio of 5.1. Although the revised merging algorithm slightly raised the *R*_1_-factor, it did not significantly change the estimated standard deviations on the geometric parameters. The three weighting schemes were also found to give more similar results, with scheme 3 down-weighting only 3.6% of the reflections. Given the similarity of the results, weighting scheme 2 was selected for the joint refinement against both X-ray and neutron data.

In this study, the neutron data collected from the mini-DAC cover a more uniform area of reciprocal space (see Section 2.2.3) and have the same quality, as judged by geometric parameters of the non-H atoms and their estimated standard deviations, compared with those obtained at the same pressure by X-ray diffraction. The theoretical maximum completeness of 83.3% that characterizes the Laue diffraction technique (Cruickshank *et al.*, 1987 ▸) is affected only marginally by the use of the high-pressure cell; the losses experienced in the experiment arise mainly from the detector edges, erasing-lamp regions and from the rejection of outliers during integration and normalization. The low data/parameter ratio is a consequence of the anisotropic refinement of the H atoms, which increases the number of parameters refined from 58 to 193.

### 3.3. Location of H atoms

**Figure 7:**
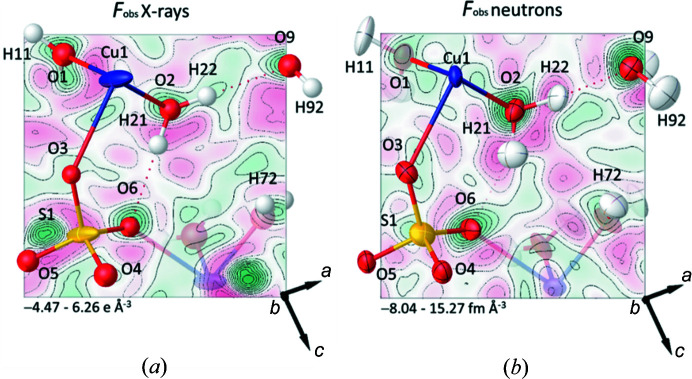
Slices in the O9—O2—O6 plane of the observed (*a*) electron and (*b*) nuclear density maps of copper sulfate pentahydrate at 0.71 GPa. Only the atoms in or above the iso-surface are labelled for clarity. Images were generated in *Olex2* (Dolomanov *et al.*, 2009 ▸). Positive and negative densities are shown in green and red, respectively.

H atoms can often be located using single-crystal X-ray diffraction data collected at ambient pressure on modern instruments. The same is not true at high pressure, generally because of low completeness and lower signal-to-noise arising from deterioration in crystal quality on compression, high background scattering and attenuation of the scattered intensity by the components of the cell. Accordingly, while the O, S and Cu atom positions in copper sulfate pentahydrate were readily determined from the X-ray data, the H atoms could not be located. By contrast, the H atoms were clearly visible in a Fourier map calculated using the neutron data. Fig. 7 ▸ shows slices in the O9—O2—O6 plane of the (*a*) X-ray and (*b*) neutron *F*_o_ scattering density iso-surfaces. For the latter data, the map clearly identifies the positions of H21 and H22, whereas for the former, the map does not reveal them at all. At the resolution of this study, the increased contrast of the strong negative scattering length of the ^1^H isotope, coming from the fact that the sample was not deuterated, aids the identification. Animation showing the progression of the *F*_o_ scattering density iso-surface through the sample, for both the X-ray and neutron experiments, are available in the supporting information.

### 3.4. XN refinement

Crystal and refinement data for the individual X-ray and neutron structures are shown in Table 4 ▸. The observed intensity data and weights were output in cif format by *Crystals* and used as input for the XN refinement in *TOPAS Academic* (version 6; Coelho, 2018 ▸). The intensities were on an absolute scale. The weights (from scheme 2) were scaled to ensure that both separate refinements yielded a goodness of fit equal to 1 (Fig. S4). The structure was modelled using the *Z*-matrix formalism, which has the same number of variables as a conventional coordinate model but the parameterization is in terms of molecular translations and rotations and intramolecular bond distances and angles. The overall position and orientation parameters, bond angles and torsions, and distances not involving H were constrained to be equal for the X-ray and neutron models. One additional parameter (*del*H) was used to account for the difference between O—H distances measured with neutron and X-ray diffraction. A commented *Topas* input file is available in the supporting information.

Initially, the primary intramolecular geometric parameters were held fixed, and only the positions and orientations of the rigid bodies were allowed to vary. All geometric parameters were refined in the final stage of the joint refinement, along with the 132 anisotropic displacement parameters. The scale factors of the neutron and X-ray data-sets were fixed to 100 and 1, respectively, as the intensities were on an absolute scale (see above) but the neutron scattering lengths embedded in *TOPAS* differ from those in *Crystals* by a factor of 10. No restraints were applied. The parameter *del*H was initially set to 0.1 Å and refined to 0.10 (3) Å.

Anisotropic displacement parameters obtained using X-ray and neutron data are known to differ (Blessing, 1995 ▸) because of differences in temperature, absorption, extinction, thermal diffuse scattering, multiple reflections and other systematic errors measured in the two experiments. The simple use of an isotropic scale factor, *q*, defined by *U*_X_^*ij*^ = *qU*_N_^*ij*^, was found to be the best fit to our data among the different approaches suggested by Blessing. The use of unrestrained anisotropic scale factors, *U*_X_^*ij*^ = *q*_*ij*_ *U*_N_^*ij*^, led to the unphysical *q*_12_ value of −0.02 (18) Å^2^, while a slight overall increase in estimated standard deviations was observed when the *q*_ij_ were restrained to be within 10% of the isotropic value.

The refinement statistics are shown in Table 4 ▸. The overall goodness of fit was close to 1, and a normal probability plot calculated using values of *w* ^1/2^(|*F*_o_|^2^ − |*F*_c_|^2^) passed through the origin (intercept = 0.07) but also showed some deviation from linearity (*r* ^2^ = 0.97; see Fig. S4). This is a sign that the residual systematic error remained in the two data-sets; the magnitudes of these errors may also be different, which is perhaps not unexpected as the corrections applied to the neutron data for cell absorption and to the X-ray data for absorption and gasket shading are substantial. In addition, the low-resolution model errors connected with the use of spherical scattering factors present in the X-ray data are absent in the neutron refinement.

**Figure 8:**
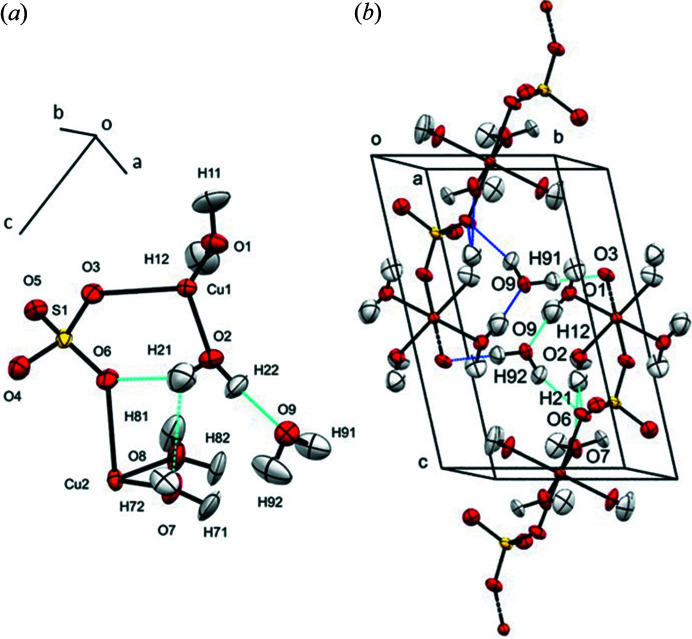
(*a*) Labelling of atoms and hydrogen-bonds (blue dotted lines) in the asymmetric unit of copper sulfate pentahydrate at 0.71 GPa. (*b*) Hydrogen bonding of the sample as viewed along a*. Ellipsoids are shown at 50% probability.

A comparison between bond lengths, angles and *U*_eq_ for the three models is shown in Table 3 ▸, with the atom labelling shown in Fig. 8 ▸(*a*). *U*_eq_ and hydrogen-bond geometries were calculated using the program *PLATON* (Spek, 2009 ▸). The standard uncertainties of both the geometric parameters and the anisotropic displacement parameters are smaller than those coming from the ‘individual’ refinements. The improvement is particularly marked in the sulfate group where the standard uncertainties in the S1—O4 and S1—O5 distances are reduced by ∼50%.

### 3.5. The crystal structure of copper sulfate pentahydrate at 0.71 GPa

The crystal structure of copper sulfate pentahydrate comprises alternating Cu(H_2_O)_4_^2+^ and SO_4_^2−^ groups. There is an additional water molecule per formula unit, so that the compound can be more accurately described as [Cu(SO_4_)(H_2_O)_4_]H_2_O. Under ambient conditions, the material crystallizes in the triclinic space group *P* 1 , with lattice parameters *a* = 5.9681, *b* = 6.1224, *c* = 10.7223 Å, α = 77.40, β = 82.35, γ = 72.67°, *V* = 364.02 Å^3^, *Z* = 2. The unit cell contains two crystallographically independent Cu^2+^ ions, which are coordinated with two pairs of crystallographically independent water molecules in equatorial positions. Axially bound SO_4_^2−^ groups, one of which is present in the asymmetric unit, form a coordination polymer. The material exhibits asymmetric tetragonal Jahn–Teller (JT) elongation in the direction of the Cu—O(sulfate), their bond lengths being approximately 19% greater than the equatorial distances with a difference between Cu1 and Cu2 axial Cu—O bonds of approximately 0.05 Å.

The unit-cell volume decreases by 1.4% between ambient pressure and 0.71 GPa, but there are no phase transitions. Most bond distances and angles vary no more than 3σ (4σ for ∠H11—O1—H12), confirming the absence of pressure sensitivity in the intramolecular geometry apart from significant reductions in the Cu2—O7 bond from 1.970 (2) to 1.951 (5) Å, and in one of the two JT bonds, Cu2—O6, from 2.440 (3) to 2.411 (7) Å. As noted by Bacon & Titterton (1975 ▸), the ∠H—O—H angles of the ligating water molecules are larger than the typical values of 105.9 and 109.7° for tetrahedrally and trigonally bound waters (see Table 3 ▸). The closest Cu⋯Cu contact along the polymer chain is shortened from 5.569 Å at ambient pressure to 5.523 Å at 0.71 GPa. The uncoordinated water molecule is involved in hydrogen bonding between chains [Fig. 8 ▸(*b*)], acting as both acceptor [O9—H91⋯O3 and O9—H92⋯O6, with a H⋯O distance of 1.809 (19) and 2.039 (19) Å, respectively] and donor (O2—H22⋯O9, 1.762 (15) Å). These distances are slightly longer at ambient pressure [1.821 (4), 2.099 (9) and 1.772 (5) Å]. While a similar comment applies to the other hydrogen bonds, the O8—H81⋯O4 and the O2—H21⋯O6 bonds elongate, the latter being part of the three-centred hydrogen bond formed within the asymmetric unit between H21, the O7 atom of a ligand water molecule and the O6 atom belonging to the sulfate group. A more detailed study of the response to further pressure application on the crystal structure of copper sulfate pentahydrate will be reported elsewhere.

## 4. Conclusions

In this study, we used single-crystal X-ray and neutron Laue data, collected on the same sample under identical conditions of pressure and temperature, to investigate the crystal structure of the triclinic salt hydrate copper(II) sulfate pentahydrate at 0.71 GPa. The results extend single-crystal XN structural refinements to the study of low-symmetry materials at high pressure.

The neutron data collected from the mini-DAC have the same quality, as judged by the precision of the geometric parameters of the non-H atoms, to those obtained at the same pressure with X-ray diffraction. The X-ray data establish the unit-cell dimensions and the positions of the non-H atoms, but suffer from low completeness because of shading of the detector by the body of the DAC. The neutron data achieve much higher completeness as well as unambiguous and accurate location of the H atoms. The use of single-crystal Laue methods makes deuteration unnecessary, as well as enabling free refinement of all parameters, including the anisotropic displacement parameters for all atoms. The sampling of reciprocal space achieved is substantially greater than in the work by Grzechnik *et al.* (2020 ▸), enabling the unconstrained refinement of the structure. The combined XN data-set yields a refined model with lower standard deviations than the individual data-sets for all parameters. The improvement in precision reaches 50% in some cases.

Joint refinement against two independent sets of data requires a model that can adapt to the different physical characteristics of each experiment. In the case of materials containing hydrogen, the most obvious of these is imposed by the determination of nuclear scattering density by neutrons and electron density by X-rays, so that the two data-sets need to be modelled with different sets of H-atom positions. A suitable model is readily built using variable-metric rigid bodies defined by *Z*-matrices, where the geometry of a molecule is defined using intramolecular bond distances, angles and torsions; six further parameters are then required to locate and orient the molecule in the unit cell. When all variables are refined, the model contains the same number of parameters as a conventional model based on atomic coordinates, but it allows the difference between the H-atom positions in the X-ray and neutron models to be encoded in just one additional parameter, the difference between the X-ray and neutron-derived O—H bond distances. A simple linear relationship can also be used to relate the anisotropic displacement parameters applied to the X-ray and neutron data.

A potentially tricky question in joint refinements is the relative weighting that should be assigned to each data-set. In this work, the crystal structure was refined against each data-set separately with the weighting schemes scaled to yield a goodness of fit of unity, placing the two weighting schemes on the same absolute scale. The weights were then used in the joint refinement and not adjusted further. The overall goodness of fit for the XN refinement was close to 1. A normal probability plot calculated using values of *w* ^1/2^(|*F*_o_|^2^ − |*F*_c_|^2^) passed through the origin, but showed some deviation from linearity, indicating the presence of residual systematic errors.

The DAC used in this work can be cooled in a closed-cycle cryostat, so that joint variable-temperature X-ray and neutron studies should also be possible down to temperatures only a few degrees above absolute zero. Use of a still-further-miniaturized cell, such as that described by Jin *et al.* (2017 ▸), would even permit cooling with open-flow cryostats such as the Oxford Cryostream. Although the focus of this study has been on diffraction, the cell is also suitable for Raman, UV-visible and other spectroscopic measurements; the CuBe alloy used to construct the cell body would potentially enable simultaneous use of a magnetic field during diffraction measurements. The main disadvantage of the cell from the point of view of neutron experiments is that the irradiated copper activates in the neutron beam and takes several days to decay to a level that permits further handling.

At only 0.71 GPa, the structural parameters of copper(II) sulfate pentahydrate are not significantly changed from those at ambient pressure, though a reduction of 0.033 Å in the Cu2—O6 JT bond points to the pressure sensitivity of this part of the crystal structure. This feature will be explored in more detail elsewhere.

The cost in neutron beam time for the work we have described was substantial (12 days), and while certain high-profile problems will warrant such a large investment of resources, the exact procedures described are unlikely to become routine. Although the cost is high, we have shown that the reward of ensuring that exactly the same sample can be studied under identical conditions using multiple techniques is sufficiently attractive that we hope that this work will prompt some further improvement to instrumentation, including beam focusing, collimation and neutron optics. Having ready access to an X-ray diffractometer adjacent to the neutron diffractometer for rapid identification of the sample reflections, sample-quality checks and unit-cell determination would optimize the use of neutron beam time. Use of a smaller DAC such as that described by Jin *et al.* (2017 ▸), which has a pressure limit of 15 GPa but is substantially smaller with lower beam attenuation than the one used here, would also enable the measurements to be conducted more quickly.

## Supplementary Material

Crystal structure: contains datablock(s) Xcuso4h2o_0.71GPa, Ncuso4h2o_0.71GPa, XNcuso4h2o_0.71GPa, Xcuso4h2o_jointR_0.71GPa. DOI: 10.1107/S2052252521010708/fs5200sup1.cif

Input files for XN refinement in Topas. DOI: 10.1107/S2052252521010708/fs5200sup2.zip

X-ray and neutron Fobs map animations. DOI: 10.1107/S2052252521010708/fs5200sup3.zip

Supporting figures and tables. DOI: 10.1107/S2052252521010708/fs5200sup4.pdf

X-ray Fobs map animation. DOI: 10.1107/S2052252521010708/fs5200sup5.mp4

Neutron Fobs map animation. DOI: 10.1107/S2052252521010708/fs5200sup6.mp4

CCDC references: 2115973, 2115974, 2115975, 2115976

## Figures and Tables

**Table 1:** Data collection strategy at ambient temperature, using the Koala Laue single-crystal diffractometer at ANSTO, for copper sulfate pentahydrate at 0.71 GPa

Mini-DAC orientation	Laue patterns	ϕ Angles (°)	Exposure per pattern (s)	Notes
1	9	6	5400	Along the cell axis – check
		−30, −18, −6, 6, 18, 30, 42	37000	Through the diamonds
		−84	37000	Through the gasket
DAC activation allowed to decay for ∼8 h, before rotating the mini-DAC 120° clockwise and re-centring				

2	11	1	5400	Along the cell axis – check
		1, −34, −24, −14, −4, 6, 16, 26, 36	39000	Through the diamonds
		−89	39000	Through the gasket
DAC activation allowed to decay for ∼8 h, before rotating the mini-DAC 120° clockwise and re-centring				

3	9	5	3600	Along the cell axis – check
		5, −31, −19, −7, 17, 29, 41	40000	Through the diamonds
		−85	40000	Through the gasket

**Table 2:** Crystallographic information for copper sulfate pentahydrate at 0.71 GPa from X-ray and neutron data collections performed at 295 K All single-crystal diffraction data were refined against *F* ^2^. All agreement factors are given as percentages. The number of reflections refers to those used in the refinement. Omitted estimated standard deviations in the intramolecular geometries indicate that those parameters were not refined in the X-ray refinement.

	Scheme 1			Scheme 2			Scheme 3				
Weighting scheme	* w =* 1/[σ^2^(|*F*_o_|^2^)+(*aP*)^2^+*bP*],			*w =* 1/[σ^2^(|*F*_o_|^2^)+(*aP*)^2^+*bP*],			*w =* (1/4(|*F*_o_|^2^)*w ’*w ’’				
	*P* = (|*F*_o_|^2^+2|*F*_c_|^2^)/3			*P* = (|*F*_o_|^2^+2|*F*_c_|^2^)/3			*w* ’ = 1/(σ^2^(|*F*_o_|)				
							*w*’’ = [1−(Δ|*F*|/6.000*|*F*_est_|)^2^]^2^				
Refinement	X	N		X	N		X	N			
		*LaueG*	*SORTAV*		*LaueG*	*SORTAV*		*LaueG*		*SORTAV*	
*a*	0.089	0.120	0.144		0.03						
*b*	2.690	22.830	0		0						
Reflections	532	958	990	532	958	990	532	958		990	
Parameters	58	193		58	193		58	193			
*R*_1_ †	6.01	16.67	23.37	6.20	17.60	23.98	6.06	16.54		23.77	
*wR*_2_ (*F* ^2^)‡	14.74	28.33	23.69	15.99	17.66	16.44	2.16	8.78		12.01	
*S* §	0.998	1.043	0.935	4.415	2.534	1.315	1.030	1.159		0.906	

Err Bonds (mean) (Å)											
Cu—O	0.011	0.009	0.008	0.009	0.008	0.008	0.004	0.005		0.006	
S—O	0.010	0.016	0.014	0.010	0.014	0.014	0.003	0.009		0.011	
O—H	–	0.02	0.017	–	0.016	0.017	–	0.011		0.014	

Err angles (mean) (°)											
O—Cu—O	0.4	0.3	0.3	0.4	0.3	0.3	0.13	0.19		0.3	
O—S—O	0.6	1.0	0.9	0.5	0.9	0.8	0.13	0.6		0.7	
Cu—O—S	0.6	0.7	0.6	0.5	0.6	0.6	0.19	0.4		0.5	
Cu—O—H	–	1.2	1.1	–	1.0	1.1	–	0.7		0.9	
H—O—H	–	1.6	1.5	–	1.0	1.4	–	0.9		1.2	

† *R*_1_ is based on *F* and data with {F_{\rm{O}}} \gt 4\sigma \left({{F_{\rm{O}}}} \right).

‡ *wR*_2_(*F* ^2^) is defined as \{ \sum {[w{{(F_{\rm o}^2 - F_{\rm c}^2)}^2}]/\sum {[w{{(F_{\rm o}^2)}^2}]{\} ^{1/2}}} }, where the sum is taken over the specified reflections and *w* is the least-squares reflection weight.

§ *S* is defined as \{ \sum {[w{{(F_{\rm o}^2 - F_{\rm c}^2)}^2}]/(n - p){\} ^{1/2}}}, where *n* and *p* are the number of reflections used in the refinement and the number of parameters, respectively.

**Table 3:** Comparison of selected bond distances (Å), angles (°) and *U*_eq_ (Å^2^) of copper sulfate pentahydrate at 0.71 GPa, obtained by neutron (N), X-ray (X) and joint (XN) refinements, with selected corresponding values at ambient conditions from the literature.

Refinement	N		X			XN				Varghese†			Bacon‡		
Bond length															
Cu1—O1	1.977 (7)		1.90 (2)			1.980 (6)				1.964 (1)			1.975 (2)		
Cu1—O2	1.966 (8)		1.971 (4)			1.972 (4)				1.971 (1)			1.974 (3)		
Cu1—O3	2.376 (6)		2.363 (6)			2.376 (6)				2.382 (1)			2.386 (2)		
Cu2—O6	2.395 (6)		2.412 (5)			2.411 (5)				2.434 (1)			2.440 (3)		
Cu2—O7	1.956 (7)		1.923 (12)			1.951 (5)				1.960 (1)			1.970 (2)		
Cu2—O8	1.939 (7)		1.932 (16)			1.938 (5)				1.932 (1)			1.945 (2)		
S1—O3	1.452 (13)		1.479 (5)			1.468 (4)				1.475 (1)			1.482 (6)		
S1—O4	1.454 (13)		1.442 (15)			1.477 (6)				1.474 (1)			1.470 (5)		
S1—O5	1.541 (13)		1.487 (16)			1.495 (7)				1.487 (1)			1.491 (3)		
S1—O6	1.486 (13)		1.471 (5)			1.475 (4)				1.476 (1)			1.476 (4)		
O1—H11	1.04 (2)		0.84			0.980 (14)				0.739 (18)			0.965 (6)		
O1—H12	0.85 (2)		0.84			0.932 (16)				0.812 (18)			0.960 (6)		
O9—H91	0.993 (19)		0.84			0.988 (14)				0.808 (17)			0.979 (5)		
O9—H92	1.004 (18)		0.84			0.972 (15)				0.802 (20)			0.914 (10)		

Angle															
H11—O1—H12	113.9 (13)		118.2			113.6 (11)				110.8 (19)			108.6 (5)		
H21—O2—H22	110.1 (12)		130.3			111.4 (10)				111.3 (17)			109.2 (6)		
H71—O7—H72	113.5 (13)		118.1			112.5 (11)				113.5 (18)			113.0 (4)		
H81—O8—H82	109.7 (11)		105.3			109.2 (10)				106.4 (19)			110.2 (4)		
H91—O9—H92	104.0 (11)		122.1			104.4 (9)				103.6 (19)			107.2 (7)		

Refinement	N	X			XN			Bacon‡				N			XN
*U*_eq_															
S1	0.027 (4)	0.034 (2)			0.0221 (10)			0.0175	H11			0.053 (5)			0.051 (4)
Cu1	0.0175 (19)	0.0396 (17)			0.0186 (7)			0.0198	H12			0.052 (6)			0.061 (5)
Cu2	0.0200 (19)	0.0365 (17)			0.0211 (7)			0.0260	H21			0.047 (5)			0.055 (5)
O1	0.029 (3)				0.0289 (16)			0.0334	H22			0.040 (4)			0.041 (4)
O2	0.029 (3)				0.0241 (15)			0.0237	H71			0.053 (5)			0.051 (4)
O3	0.026 (2)				0.0251 (11)			0.0272	H72			0.041 (4)			0.036 (4)
O4	0.029 (2)				0.0285 (14)			0.0397	H81			0.050 (5)			0.047 (5)
O5	0.028 (2)				0.0283 (14)			0.0372	H82			0.045 (5)			0.044 (4)
O6	0.028 (2)				0.0283 (13)			0.0187	H91			0.043 (5)			0.044 (4)
O7	0.031 (3)				0.0310 (16)			0.0439	H92			0.064 (6)			0.060 (5)
O8	0.033 (3)				0.0334 (17)			0.0625							
O9	0.027 (3)				0.0261 (16)			0.0331							

Refinement				XN							Bacon‡				
Hydrogen bond				H⋯*A*			∠*D*—H⋯*A*				H⋯*A*			∠D—H⋯*A*	
O1—H11 O3				2.493 (15)			124.6 (11)				2.510 (5)			128.3 (4)	
O1—H11⋯O5				1.870 (18)			168.0 (14)				1.913 (7)			165.3 (5)	
O1—H12⋯O9				1.79 (2)			171.0 (17)				1.835 (7)			168.6 (5)	
O2—H21⋯O6 in asymmetric unit				1.931 (16)			152.6 (14)				1.897 (6)			153.9 (6)	
O2—H21⋯O7 in asymmetric unit				2.573 (16)			122.3 (11)				2.601 (6)			120.6 (6)	
O2—H22⋯O9				1.762 (15)			174.9 (18)				1.772 (5)			172.3 (5)	
O7—H71⋯O4				1.716 (19)			173.4 (17)				1.745 (4)			174.7 (3)	
O7—H72⋯O5				1.77 (2)			170 (2)				1.808 (5)			169.1 (5)	
O8—H81 ⋯ O4				1.73 (2)			173.5 (16)				1.712 (5)			175.8 (4)	
O8—H82 ⋯ O5				1.72 (2)			166.4 (17)				1.759 (4)			167.7 (5)	
O9—H91 ⋯ O3				1.809 (19)			166 (2)				1.821 (4)			168.1 (4)	
O9—H92 ⋯ O6				2.039 (19)			160.8 (16)				2.099 (9)			161.6 (7)	

† Calculated primary intramolecular geometries from the X-ray coordinates of Varghese & Maslen (1985 ▸) of copper sulfate pentahydrate at ambient pressure and 298 K.

‡ Calculated primary intramolecular geometries from the neutron coordinates of Bacon & Titterton (1975 ▸) of copper sulfate pentahydrate at ambient pressure and 298 K.

**Table 4:** Crystallographic information for copper sulfate pentahydrate at 0.71 GPa X-ray and neutron data collections were performed at ambient temperature. Refinement of the crystal structure was performed using weighting scheme 2. For the XN refinement the *R*-factors quoted are based on *F* ^2^ and all data.

CCDC Deposition Number		2115973		2115974	
Pressure (GPa)		0.71		0.71	
Crystal data					
Crystal system, space group		Triclinic, *P* 1		Triclinic, *P* 1	
Parameters					
*a* (Å)		5.9523 (12)		Set equal to the X-ray values	
*b* (Å)		6.1001 (13)			
*c* (Å)		10.6470 (7)			
α (°)		77.308 (10)			
β (°)		82.353 (10)			
γ (°)		72.640 (11)			
*V* (Å^3^)		359.02 (11)			
ρ (g cm^−3^)		2.310			
Radiation type		Mo *K*α, λ = 0.71073 Å		Neutrons, λ = 0.78 – 3.50 Å	
Crystal size (mm)		0.40 × 0.22 × 0.20		0.40 × 0.22 × 0.20	

Data collection					
Diffractometer		Agilent SuperNova		Koala	
Absorption correction		Multi-scan		N/A	
No. of measured, independent and observed [*I* > 4σ(*I*)] reflections		3200, 552, 521		9163, 1147, 565	
Reflections used in refinement		532		990	
*R*_int_		0.054		0.145	
θ_min_ (°), θ_max_ (°)		3.934, 30.474		4.2, 72	
Completeness (%)		24		70	
*d*_min_		0.7		0.8	

Refinement					
*R*[*F* ^2^ > 4σ(*F* ^2^)], *wR*_2_ (*F* ^2^), *S*		0.062, 0.160, 0.998		0.2398, 0.1651, 1.000	
Unique reflections/parameters		9.2		5.1	
No. of restraints		0		0	
Δρ_max_, Δρ_min_ (e Å^−3^/ nuclear Å^−3^)		0.46, −0.56		2.69, −2.65	

XN joint refinement					
R_Total	0.2085	Rw_Total	0.1692	GooF_Total	1.01
R_Neutron	0.2470	Rw_Neutron	0.1756	GooF_Neutron	1.06
R_X-ray	0.1112	Rw_X-ray	0.1585	GooF_X-ray	1.17
Unique reflections/parameters		7.8			
No. of restraints		0			
Additional parameters		*del*H	0.10 (3)	*q*[*U*_X_^ij^=*qU*_N_^ij^]	1.01 (3)
