# Supplementary figures and images for: Use of a miniature diamond-anvil cell in a joint X-ray and neutron high-pressure study on copper sulfate pentahydrate
Source: IUCrJ. 2021 Nov 20;9(Pt 1):73–85. doi: 10.1107/S2052252521010708 (PMC8733890; doi:10.1107/S2052252521010708)

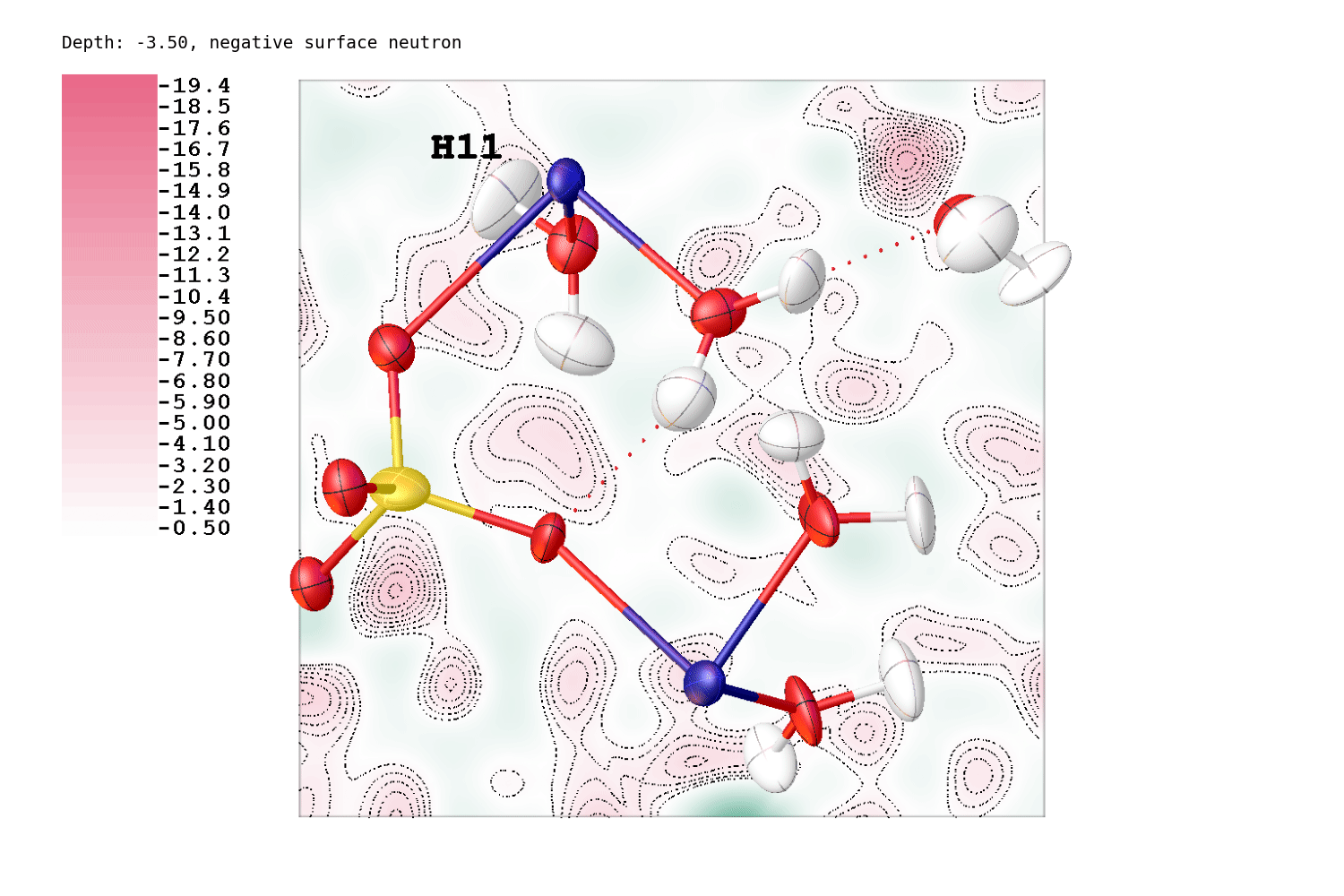

Supplement: Supplementary file 3 [file m-09-00073-sup3.zip › movie-neutron.gif]

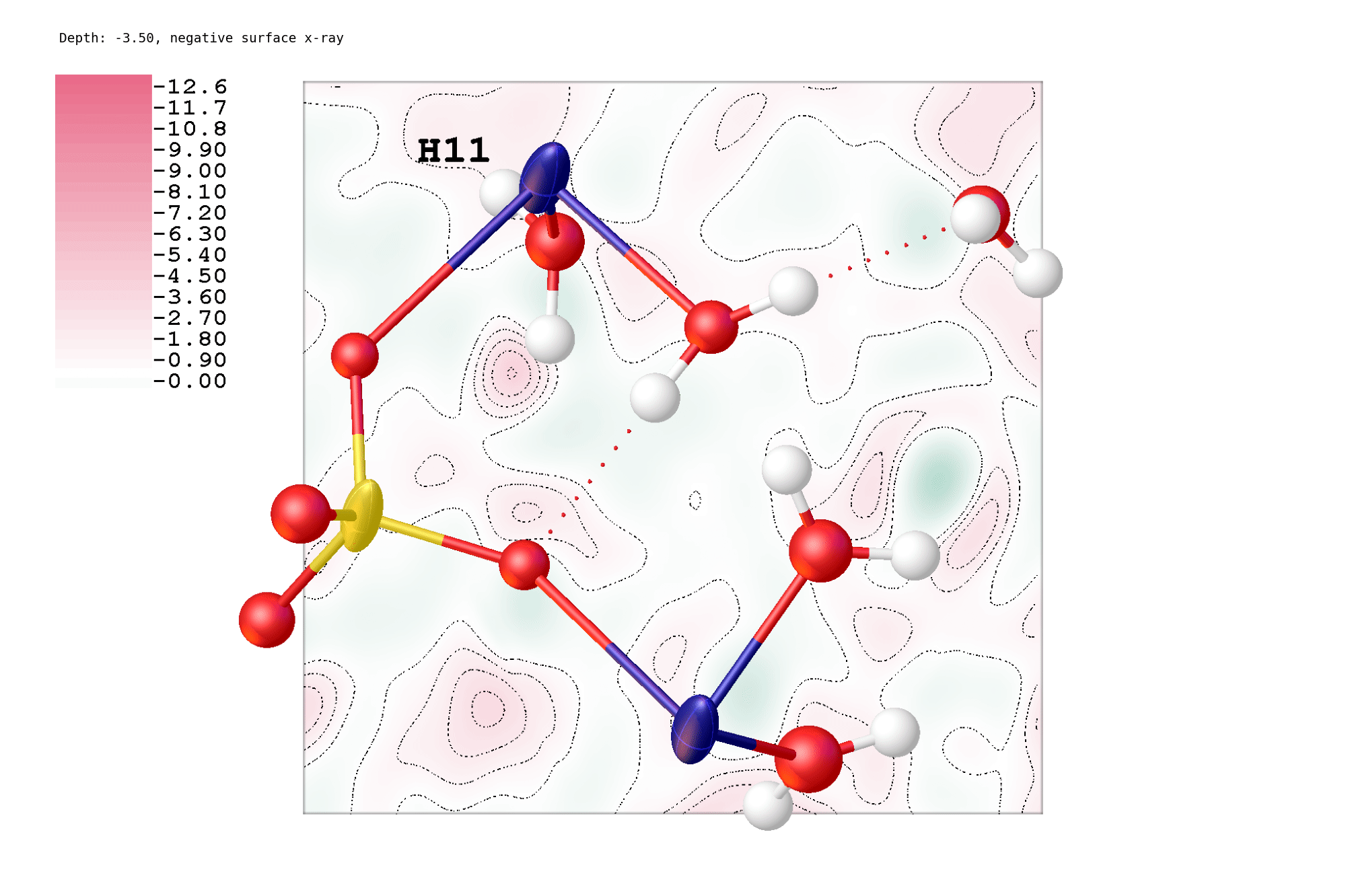

Supplement: Supplementary file 3 [file m-09-00073-sup3.zip › movie-Xray.gif]
