# Supplementary material for: Use of a miniature diamond-anvil cell in a joint X-ray and neutron high-pressure study on copper sulfate pentahydrate
Source: IUCrJ. 2021 Nov 20;9(Pt 1):73–85. doi: 10.1107/S2052252521010708 (PMC8733890; doi:10.1107/S2052252521010708)
Supplement: Supplementary file 4 [file m-09-00073-sup4.pdf]

IUCrJ

Volume 9 (2022)

Supporting information for article:

Use of a Miniature Diamond-anvil Cell in a Joint X-ray and Neutron High-pressure Study on Copper Sulfate Pentahydrate

Giulia Novelli, Konstantin V. Kamenev, Helen E. Maynard-Casely, Simon Parsons and Garry J. McIntyre

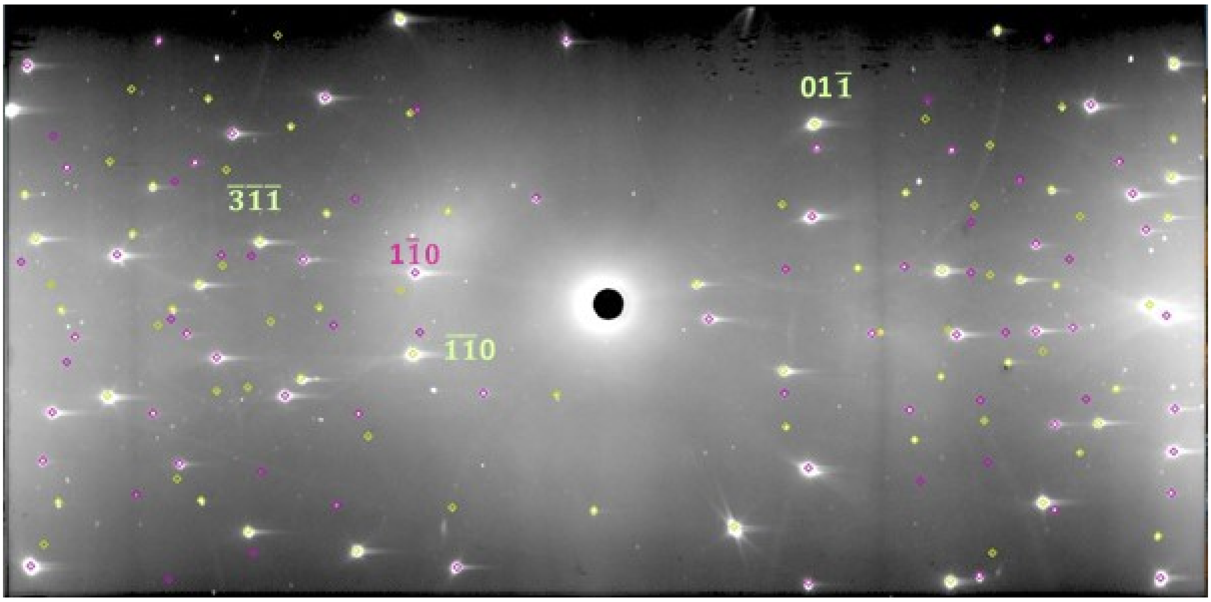

Figure S1 Laue diffraction pattern of copper sulfate pentahydrate recorded at 0.71 GPa, $\phi = -30°$ using the mini-DAC. The two sets of diamonds were indexed as twins in *LaueG* with their calculated spots being green and purple, respectively. The labelled diamond reflections are those mentioned in the caption to Fig. 3.

Table S1 Integration parameters during processing of the neutron data collected on Koala.

**Parameter**	Value
Target peak fraction	0.8
Area multiplier for peak ellipse	4
Peak fraction uncertainty	25
Neighbour overlap tolerance	10
Model ellipses in middle zone	10
Model ellipses in outer zones	10
Cutoff level of contour ellipse	0.3
Min. fill fraction for contour	0.3
Min. d-spacing for hkls	0.8
Min. and max. wavelength for hkls	0.78 – 99.9

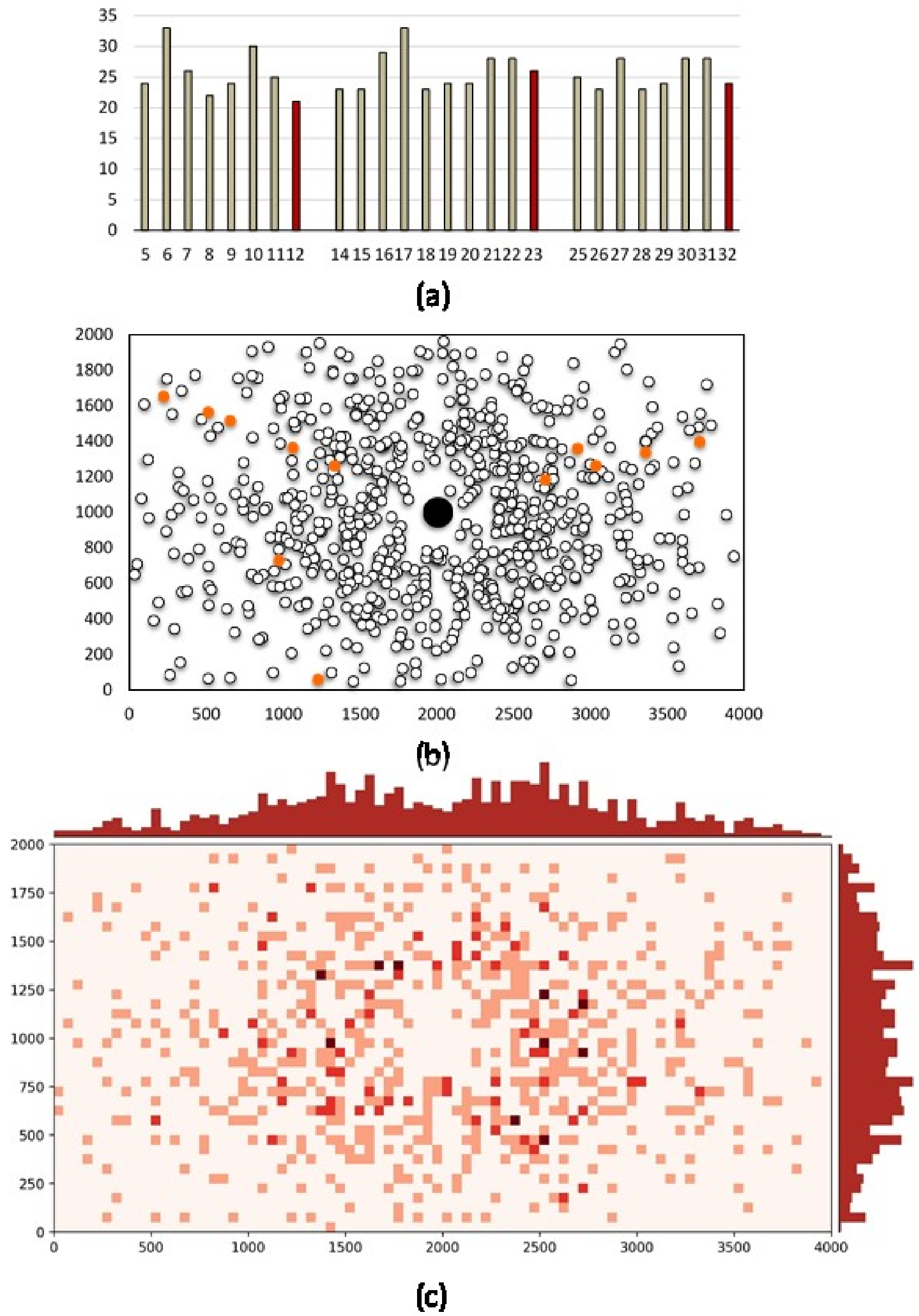

Figure S2 (a) Number of rejected reflection distributed among the Laue patterns, with the red ones highlighting the patterns taken with the incident neutron beam passing through the gasket. (b) un-merged rejected reflections distributed across the image-plate detector, for all the collected patterns, the orange spots indicating the location of the reflections belonging to the 259 reflection and its symmetry equivalents. (c) Distribution of the same rejected reflections as a thermal plot with respective histograms along the length and width of the image-plate detector.

Table S2 The 259 reflection and its symmetry equivalents listed based on their appearance in the Laue patterns, un-merged and merged intensities produced using the program LaueG, with

their respective estimated standard deviations, and the calculated intensity from the structural refinement performed with scheme 3.

h	*k*	*l*	Pattern	I	σ	Notes	Average I	σ	Scheme 3	weight
2	5	9	21	405.2	318.3	Close to the exit path of the neutron beam	511.3	11.1	247.31	0
2	5	9	27	677.3	211.6					
2	5	9	11	293.5	67.9	Pattern edge				
**2**	**5**	**9**	**23**	**699.4**	**119.0**	Area of highly-varying background				
2	5	9	20	401.9	47.3					
2	5	9	28	637.0	42.0					
2	5	9	14	585.5	28					
2	5	9	25	443.3	21					
2	5	9	18	629.2	32.9					
2	5	9	17	551.2	27.2					
2	5	9	29	376.8	34.9					
**−2**	**−5**	**−9**	**32**	**634.6**	**86**	Area of highly-varying background				

Image 32

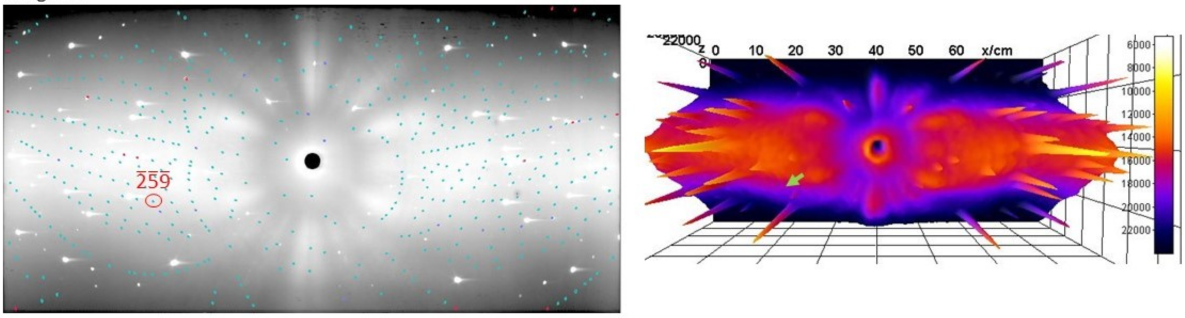

Image 23

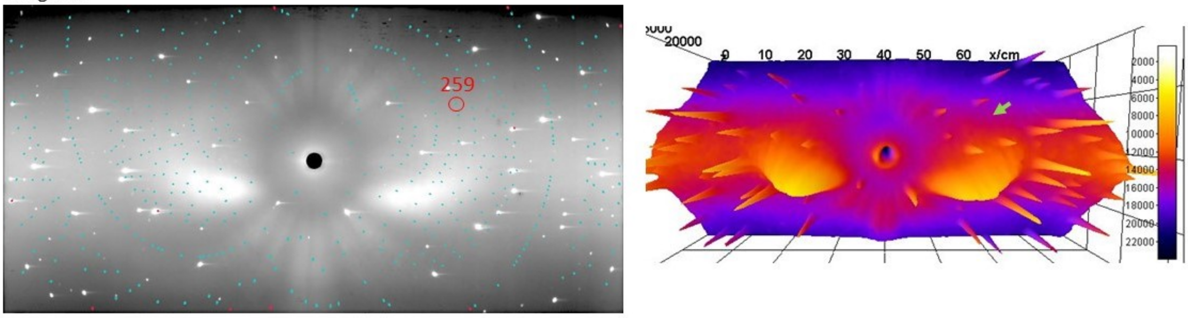

Figure S3 Laue patterns with their 3D thermal plots showing the outliers in proximity of areas of highly-varying background. The background varies from pattern t pattern and seems affected by both the DAC-orientation and the diamonds.

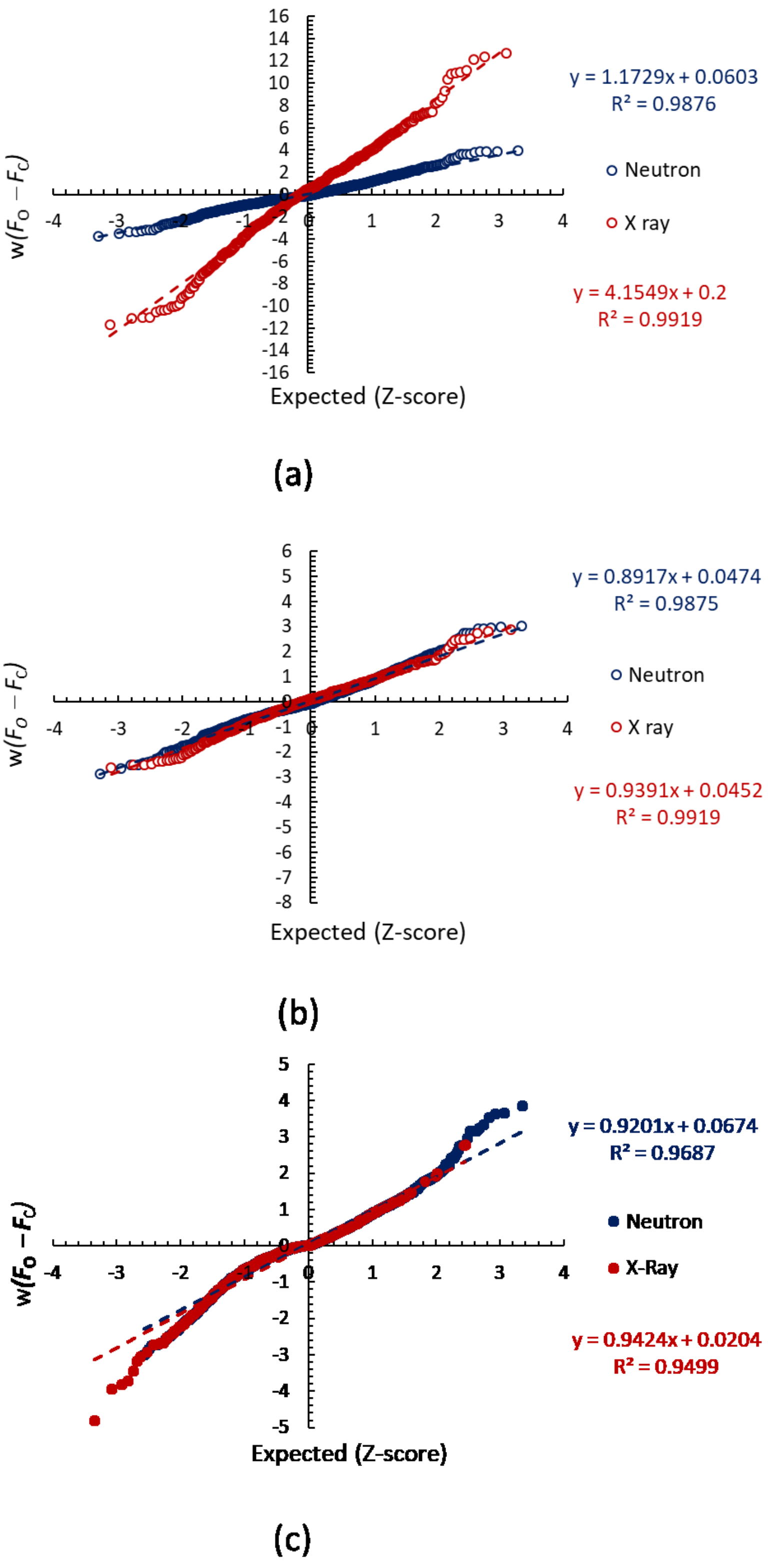

Figure S4 Normal probability plots of the 'individual' X-ray and neutron refinements with scheme 2, before (a) and after (b) scaling of the weights to 1, and for the 'X-N' structure refinement (c).

Table S3 Comparison of bond distances (Å) and angles (°) of copper sulfate pentahydrate at 0.71 GPa, obtained with neutron (**N**), X-ray (**X**), and joint (**X-N**) refinements, with selected ambient-conditions corresponding values from the literature.

Refinement	N	X	X-N	Varghese & Maslen (1985)[a]	Bacon & Titterton (1975)[b]
**Bond length**					
Cu1–O1	1.977(7)	1.90(2)	1.980(5)	1.964(1)	1.975(2)
Cu1–O2	1.966(8)	1.972(4)	1.972(4)	1.971(1)	1.974(3)
Cu1–O3	2.376(6)	2.363(6)	2.376(6)	2.382(1)	2.386(2)
Cu2–O6	2.395(6)	2.412(5)	2.411(7)	2.434(1)	2.440(3)
Cu2–O7	1.956(7)	1.923(12)	1.951(5)	1.960(1)	1.970(2)
Cu2–O8	1.939(7)	1.932(16)	1.938(5)	1.932(1)	1.945(2)
S1–O3	1.452(13)	1.479(5)	1.468(4)	1.475(1)	1.482(6)
S1–O4	1.454(13)	1.442(15)	1.477(6)	1.474(1)	1.470(5)
S1–O5	1.541(13)	1.487(16)	1.495(7)	1.487(1)	1.491(3)
S1–O6	1.486(13)	1.471(5)	1.475(4)	1.476(1)	1.476(4)
O1–H11	1.04(2)	0.84	0.980(14)	0.739(18)	0.965(6)
O1–H12	0.85(2)	0.84	0.932(16)	0.812(18)	0.960(6)
O2–H21	0.90(2)	0.84	0.930(15)	0.808(16)	0.966(8)
O2–H22	1.006(16)	0.84	0.976(12)	0.774(16)	0.983(5)
O7–H71	0.984(19)	0.84	0.972(11)	0.775(18)	0.971(4)
O7–H72	0.956(17)	0.84	0.972(13)	0.772(14)	0.959(5))
O8–H81	0.933(17)	0.84	0.931(14)	0.747(19)	0.963(5)
O8–H82	0.994(19)	0.84	0.990(11)	0.780(15)	0.969(5)
O9–H91	0.993(19)	0.84	0.988(14)	0.808(17)	0.979(5)
O9–H92	1.004(18)	0.84	0.972(15)	0.802(20)	0.914(10)
**Angle**					
O1–Cu1–O2	86.8(3)	87.7(4)	88.06(19)	88.24(3)	88.44(11)
O1–Cu1–O3	90.7(3)	92.8(5)	91.1(2)	91.81(3)	91.73(9)
O2–Cu1–O3	91.0(3)	91.93(19)	91.86(16)	91.41(3)	91.39(11)
O6–Cu2–O7	88.4(3)	88.1(3)	88.4(2)	88.06(4)	88.20(9)
O6–Cu2–O8	86.3(3)	86.8(3)	86.6(2)	86.69(3)	86.72(10)
O7–Cu2–O8	89.5(3)	88.4(6)	89.3(2)	89.50(4)	89.73(11)
O3 –S1–O4	111.2(8)	110.2(7)	110.2(4)	110.25(4)	110.1(3)
O3–S1–O5	107.4(7)	109.1(6)	108.2(3)	108.44(4)	108.5(3)
O3–S1–O6	110.3(8)	109.2(3)	109.7(2)	109.32(4)	109.0(3)
O4 –S1–O5	107.7(8)	108.1(6)	108.8(9)	108.82(4)	109.1(3)
O4–S1–O6	114.0(9)	111.0(6)	111.7(6)	111.07(5)	111.7(3)
O5–S1–O6	105.8(8)	109.2(7)	108.2(6)	108.88(4)	108.5(2)
Cu1–O3–S1	132.1(6)	132.3(3)	132.0(3)	132.56(4)	132.62(16)
Cu2–O6–S1	138.8(6)	139.6(4)	139.1(4)	139.25(5)	138.8(3)
Cu1–O1–H11	114.0(10)	117.3	115.6(10)	115.8(15)	117.8(3)
Cu1–O1–H12	115.7(9)	108.1	113.5(11)	113.4(14)	115.3(4)
Cu1–O2–H21	115.1(9)	96.6	113.6(10)	113.3(13)	112.4(3)
Cu1–O2–H22	118.0(9)	118.6	118.4(12)	118.8(13)	120.3(3)
Cu2–O7–H71	120.5(10)	118.5	121.5(8)	121.0(13)	120.4(3)
Cu2–O7–H72	125.5(10)	123.4	125.6(13)	124.7(13)	126.2(3)
Cu2–O8–H81	125.6(9)	122.3	126.2(7)	127.0(14)	124.4(3)
Cu2–O8–H82	124.7(9)	132.2	124.6(12)	126.6(14)	125.4(3)
H11–O1–H12	113.9(13)	118.2	113.6(11)	110.8(19)	108.6(5)
H21–O2–H22	110.1(12)	130.3	111.4(10)	111.3(17)	109.2(6)
H71–O7–H72	113.5(13)	118.1	112.5(11)	113.5(18)	113.0(4)
H81–O8–H82	109.7(11)	105.3	109.2(10)	106.4(19)	110.2(4)
H91–O9–H92	104.0(11)	122.1	104.4(9)	103.6(19)	107.2(7)

[a] Calculated primary intramolecular geometries, from the X-ray coordinates by Varghese and Maslen (1985), of copper sulfate pentahydrate at ambient pressure and 298 K.

[b] Similar selected figures from the neutron coordinates of Bacon and Titterton (1975).
